# Interpreting vaccine-associated survival differences in immune checkpoint inhibitor therapy

**DOI:** 10.1073/pnas.2621501123

**Published:** 2026-08-31

**Authors:** René Karadakic, Nancy L. Keating, Michael L. Barnett

**Affiliations:** ^a^ Department of Management, Technology, and Economics, ETH Zürich, Zürich 8092, Switzerland; ^b^Department of Health Care Policy, Harvard Medical School, Boston, MA 02115; ^c^Department of Health Services, Policy & Practice, Brown University School of Public Health, Providence, RI 02903

**Keywords:** cancer immunotherapy, vaccination, patient survival

## Abstract

Recent studies proposed that SARS-CoV-2 mRNA vaccination may sensitize tumors to immune checkpoint inhibitor (ICI) therapy through immune modulation. Using 100% national Medicare fee-for-service claims data, we evaluated whether survival differences among vaccinated patients receiving ICIs were specific to mRNA vaccines. Among 121,676 beneficiaries initiating systemic anticancer therapy, including 10,824 initiating ICIs, vaccination near treatment initiation was associated with lower mortality across mRNA COVID-19, adenoviral COVID-19, and influenza vaccines. We found that survival associations and formal vaccine–ICI interaction effects were similar across vaccine platforms. These findings suggest that currently available population-level survival data do not isolate an mRNA-specific tumor-sensitization mechanism.

Immune checkpoint inhibitors (ICIs) have transformed treatment for multiple malignancies by enhancing antitumor immune responses (1, 2). Recently, Grippin et al. reported that receipt of a SARS-CoV-2 mRNA vaccine near initiation of ICI therapy was associated with substantially improved survival among patients with melanoma and non–small-cell lung cancer and interpreted these findings as evidence that clinically available mRNA vaccines may sensitize tumors to immune checkpoint blockade through immune modulation (3). Experimental analyses in mouse models and human tissue further suggested activation of innate immune pathways potentially capable of augmenting antitumor immunity (3). If such effects were specific to mRNA vaccine platforms, the implications would extend beyond infection prevention and suggest that non–tumor-specific mRNA vaccines may function as broadly available immunologic adjuvants in cancer therapy. Previous observational studies have also reported improved outcomes among vaccinated patients receiving ICIs (4), whereas more recent work has questioned whether these associations reflect vaccine-specific biological effects or instead arise from selection bias and other sources of confounding (5).

Despite these differing interpretations, whether currently available observational survival data uniquely support an mRNA-specific tumor-sensitization mechanism remains uncertain. In observational studies, evidence for treatment-specific synergy requires demonstrating that associations differ meaningfully across treatment groups rather than comparing point estimates alone. Apparently larger survival associations among vaccinated ICI recipients may reflect true biological interaction, but they may also arise from statistical uncertainty, limited sample size, or differences in patient populations. Distinguishing between these explanations requires formal interaction testing and comparison across vaccine platforms. Comparison across vaccine platforms is particularly informative because adenoviral COVID-19 vaccines provide protection against SARS-CoV-2 infection while relying on a biological mechanism distinct from that of mRNA vaccines. If improved survival among vaccinated ICI recipients reflects an mRNA-specific tumor-sensitization effect, survival associations would be expected to differ meaningfully between mRNA and adenoviral vaccines. Conversely, similar associations across platforms would be more consistent with mechanisms not unique to mRNA vaccination. Here, we directly evaluate this hypothesis by comparing survival associations across mRNA COVID-19, adenoviral COVID-19, and influenza vaccines and by formally testing whether vaccine-associated survival differs between patients receiving ICIs and those receiving other systemic anticancer therapies.

## Results

We analyzed data for 100% of Medicare fee-for-service beneficiaries in the United States who initiated systemic anticancer therapy between September 2020 and June 2021, including 10,824 patients initiating ICI therapy. Vaccination exposure was defined as receipt of an mRNA COVID-19 vaccine (Pfizer-BioNTech or Moderna), adenoviral COVID-19 vaccine (Janssen), or influenza vaccine within 100 d of anticancer treatment initiation. Overall survival was measured from the date of systemic anticancer therapy initiation. We estimated Kaplan–Meier curves, Cox proportional hazards models, and formal vaccine–ICI interaction models. Full methodological details are provided in *Materials and Methods* and *SI Appendix*.

**Fig. 1.**
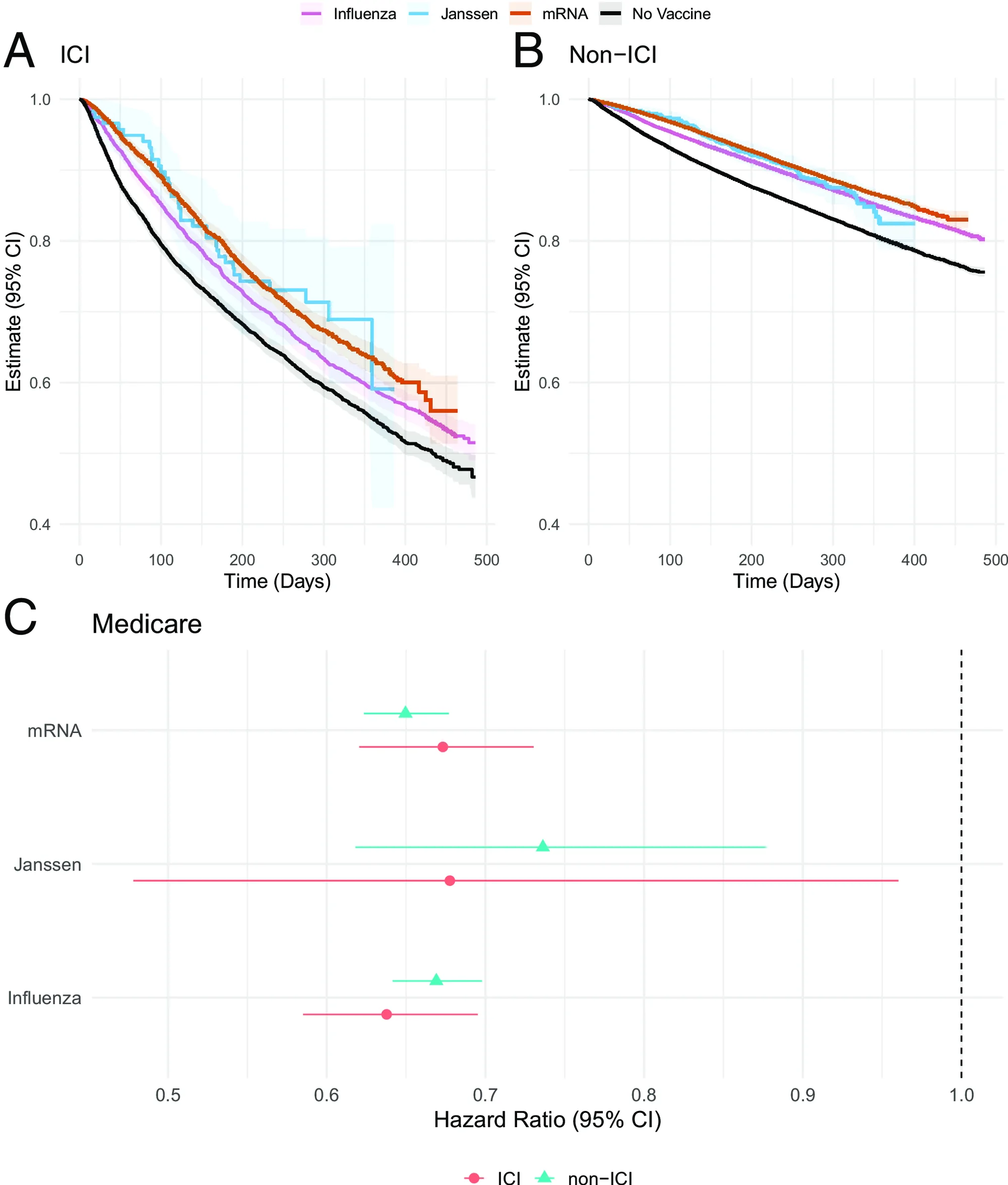
Vaccine-associated survival among ICI and non-ICI chemotherapy recipients. (*A*) Unadjusted Kaplan–Meier survival curves with 95% CIs for ICI recipients, stratified by vaccine type. (*B*) Unadjusted Kaplan–Meier survival curves with 95% CIs for non-ICI recipients, stratified by vaccine type. (*C*) Adjusted hazard ratios from Cox proportional-hazards models comparing each vaccine type to the unvaccinated group, estimated separately for ICI and non-ICI cohorts.

Among 121,676 Medicare beneficiaries initiating systemic anticancer therapy, vaccination near treatment initiation was associated with improved survival among both ICI and non-ICI recipients (Fig. 1). Among ICI recipients, adjusted Cox proportional hazards models demonstrated lower mortality relative to no vaccination for mRNA vaccination (hazard ratio [HR], 0.67; 95% CI, 0.62 to 0.73), adenoviral vaccination (HR, 0.68; 95% CI, 0.48 to 0.96), and influenza vaccination (HR, 0.64; 95% CI, 0.58 to 0.70). Direct comparison of mRNA and adenoviral vaccination showed no statistically significant difference in survival (HR, 1.16; 95% CI, 0.80 to 1.68).

Among non-ICI recipients, similar vaccine-associated survival improvements were observed for mRNA vaccination (HR, 0.65; 95% CI, 0.62 to 0.68), adenoviral vaccination (HR, 0.74; 95% CI, 0.62 to 0.88), and influenza vaccination (HR, 0.67; 95% CI, 0.64 to 0.70) relative to no vaccination (Fig. 1*B*). Kaplan–Meier curves similarly demonstrated the lowest survival among unvaccinated patients and relatively better survival among vaccinated groups in both ICI and non-ICI cohorts (Fig. 1 *A* and *B*).

**Fig. 2.**
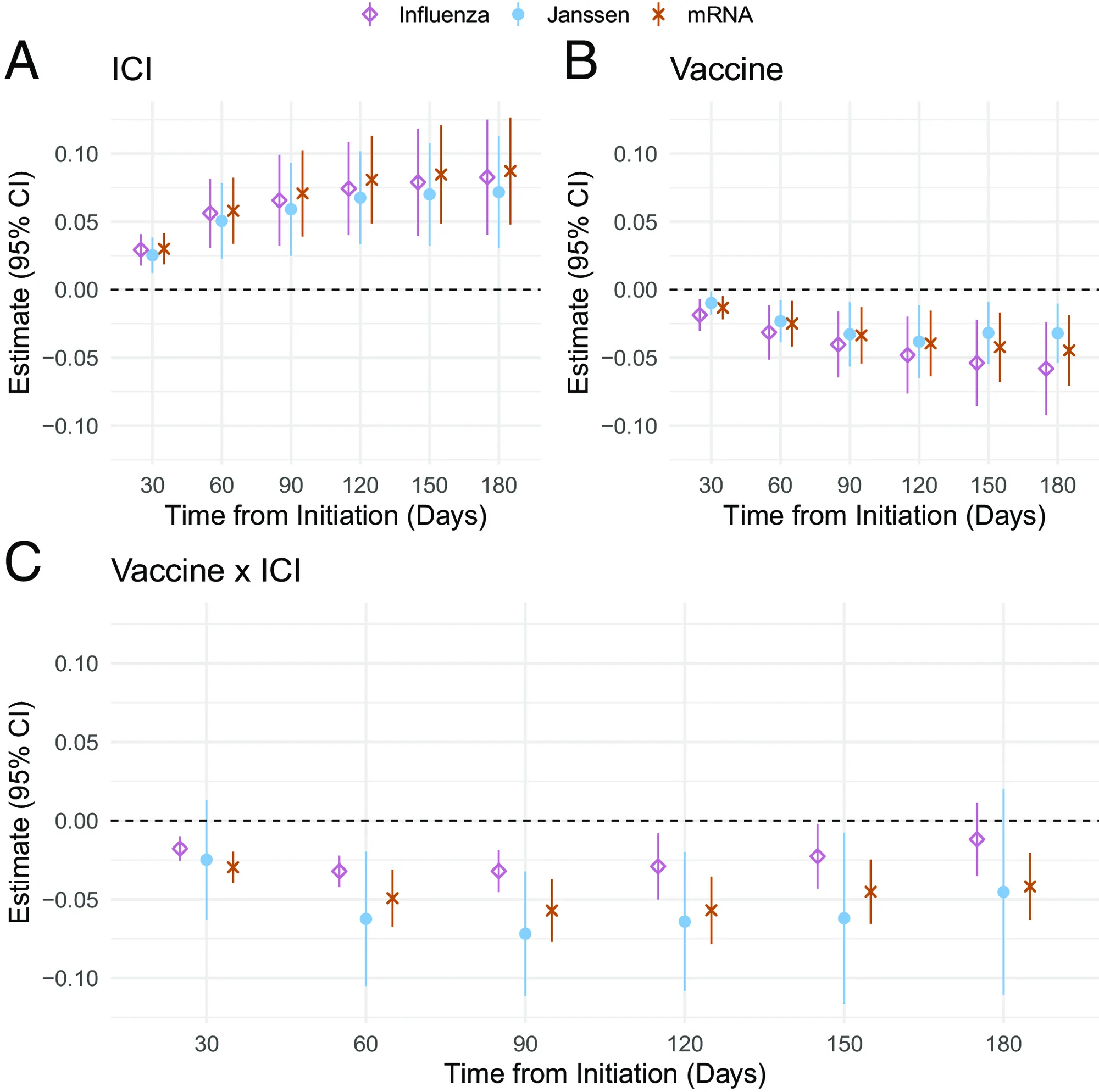
Interaction effects between vaccine receipt and ICI therapy on short-term mortality. Coefficients are derived from linear probability models estimating the association between ICI therapy, vaccine receipt, and their interaction for binary outcomes of death within X days of chemotherapy initiation. Separate regressions were estimated for each vaccine type: influenza (purple, diamonds), adenoviral Janssen (blue, circles), and mRNA (orange, crosses). Panel (*A*) reports the coefficient on ICI, representing the mortality difference between ICI and non-ICI recipients among unvaccinated patients. Panel (*B*) reports the coefficient on Vaccine, representing the mortality difference between vaccinated and unvaccinated patients among non-ICI recipients. Panel (*C*) shows the Vaccine × ICI interaction term, which indicates whether the effect of vaccination differs for ICI recipients relative to non-ICI recipients.

Our analysis demonstrated that vaccination was associated with lower mortality across both ICI and non-ICI cohorts (Fig. 2). Interaction estimates were negative for all vaccine types, indicating an additional mortality reduction associated with vaccination among ICI recipients relative to patients receiving other systemic anticancer therapies; however, the magnitude of these interaction effects was similar across vaccine platforms. At 120 d, the additional mortality reduction associated with vaccination among ICI recipients relative to patients receiving other systemic anticancer therapies was 5.7 percentage points for mRNA vaccination (95% CI, −7.8 to −3.6) and 6.4 percentage points for adenoviral vaccination (95% CI, −10.8 to −2.0). Interaction estimates for influenza vaccination were also negative, although smaller in magnitude than those observed for COVID-19 vaccines. Thus, although vaccination was associated with greater mortality reductions among ICI recipients, these additional benefits were not specific to mRNA vaccination. These findings are therefore consistent with the possibility that vaccination-associated survival differences may be greater among patients receiving ICIs, but they do not support the interpretation that such associations are uniquely attributable to mRNA-mediated tumor sensitization.

## Discussion

This large population-level analysis does not support an mRNA-specific tumor-sensitization mechanism among ICI recipients. Vaccination near ICI initiation was consistently associated with improved short-term survival, and formal interaction analyses suggested an additional survival benefit. However, similar interaction effects were observed across mRNA, adenoviral, and influenza vaccines, indicating that the observed interaction is unlikely to be unique to mRNA vaccine platforms. Because adenoviral COVID-19 vaccination was uncommon in this national cohort, the corresponding estimates are necessarily less precise despite using 100% Fee-for-Service Medicare data.

Importantly, these findings do not contradict experimental evidence that vaccination may influence immune pathways relevant to antitumor immunity. Rather, they suggest that observational survival data do not distinguish between an mRNA-specific mechanism and broader explanations operating across vaccine platforms. Previous observational studies have reported improved outcomes among vaccinated patients receiving ICIs and proposed that vaccination may augment antitumor immunity (4), whereas more recent work has emphasized the potential role of selection bias and other sources of confounding (5). Our results are broadly consistent with this literature while extending it by directly comparing vaccine platforms and formally evaluating vaccine-by-ICI interaction effects. Several mechanisms could plausibly contribute to the observed associations, including protection against infection-related morbidity and mortality (6), immune activation that is not unique to mRNA vaccines, interactions between vaccination and immune checkpoint blockade extending beyond mRNA platforms, or residual confounding related to patient selection and clinical practice patterns (7, 8). A limitation of our study is that Medicare claims capture all-cause but not cancer-specific mortality. Consequently, we cannot determine whether the observed survival differences reflect reduced infection-related mortality, improved cancer outcomes, or both. Moreover, the early separation of the survival curves is more consistent with vaccine-mediated protection against COVID-19 or influenza than with changes in cancer progression alone. Notably, a similarly early divergence is also evident in the study by Grippin et al. (3).

More broadly, our findings illustrate the importance of formal interaction testing when interpreting subgroup-specific survival associations in observational studies. As illustrated by Grippin et al. (3), apparently larger treatment effects in one subgroup than another do not, by themselves, establish biological synergy. Distinguishing treatment-specific mechanisms from statistical uncertainty requires direct comparison of interaction effects and, when possible, comparison across alternative interventions that provide a natural test of the proposed mechanism. Large-scale population data can therefore complement translational and mechanistic studies by helping distinguish whether observed clinical benefits are specific to a proposed biological mechanism or reflect broader effects operating across patient populations and interventions.

## Materials and Methods

We conducted a retrospective cohort study using deidentified Medicare claims for 100% of Medicare beneficiaries in the United States with cancer who initiated infused or injected systemic anticancer therapy between September 1, 2020, and June 30, 2021. The data were accessed through the Centers for Medicare & Medicaid Services (CMS) Virtual Research Data Center (VRDC) under a Data Use Agreement. Because the study used deidentified secondary data and did not involve identifiable human participants, institutional review board approval and informed consent were not required. Anticancer therapy was identified using Healthcare Common Procedure Coding System (HCPCS) codes for immune checkpoint inhibitors (ICIs), cytotoxic chemotherapy, hormonal therapy, and targeted therapies. Patients were required to have no infused or injected systemic anticancer therapy during the prior 365 d. Follow-up extended through December 31, 2021. The non-ICI cohort was restricted to the same cancer types represented among ICI recipients.

Vaccination exposure was defined using Medicare claims for SARS-CoV-2 mRNA vaccination (Pfizer-BioNTech or Moderna), adenoviral SARS-CoV-2 vaccination (Janssen), or influenza vaccination occurring within 100 d of systemic anticancer therapy initiation, i.e., 100 d before or after treatment initiation. Separate indicators were created for each vaccine type; thus, patients receiving more than one vaccine type within the exposure window contributed to each corresponding vaccine-specific analysis. Vaccination was analyzed separately for mRNA COVID-19, adenoviral COVID-19, and influenza vaccines. To enable direct comparison of the two COVID-19 vaccine platforms, recipients of Janssen vaccination were excluded from analyses of mRNA vaccination, and recipients of mRNA vaccination were excluded from analyses of Janssen vaccination. Influenza vaccination was analyzed independently and was not used as an exclusion criterion for the COVID-19 vaccine analyses. The primary outcome was all-cause mortality measured from the date of systemic anticancer therapy initiation.

We estimated Kaplan–Meier survival curves separately for ICI and non-ICI recipients stratified by vaccination status. Cox proportional hazards models were used to estimate adjusted hazard ratios comparing vaccinated and unvaccinated patients. Models adjusted for age; race and ethnicity; disability status; continuous Medicare fee-for-service enrollment; categorical months of dual eligibility and low-income subsidy; 27 chronic condition indicators from the Chronic Conditions Data Warehouse; cancer type fixed effects; and month-by-year fixed effects for treatment initiation.

To directly assess whether vaccination effects differed between ICI and non-ICI recipients, we estimated separate linear probability models for cumulative mortality within 30, 60, 90, 120, 150, and 180 d following treatment initiation. These models adjusted for the same covariates as the Cox proportional hazards models. Models included indicators for ICI therapy, vaccination status, and their interaction. Interaction coefficients therefore captured whether the association between vaccination and mortality differed among patients receiving ICIs relative to non-ICI systemic therapy. Additional cohort construction details and coding definitions are provided in *SI Appendix*.

## Supplementary Material

Appendix 01 (PDF)

## Data Availability

The Medicare claims data used in this study were obtained under a Data Use Agreement with the Centers for Medicare & Medicaid Services (CMS) and are not publicly available. De-identified analytic code is available from the corresponding author upon reasonable request.
