# Supplementary material for: Interpreting vaccine-associated survival differences in immune checkpoint inhibitor therapy
Source: Proc Natl Acad Sci U S A. 2026 Aug 31;123(36):e2621501123. doi: 10.1073/pnas.2621501123 (PMC13552672; doi:10.1073/pnas.2621501123)
Supplement: Supplementary file 1 [file pnas.2621501123.sapp.pdf]

Supporting Information for

Interpreting Vaccine-Associated Survival Differences in Immune Checkpoint Inhibitor Therapy

René Karadakic, Nancy L. Keating and Michael L. Barnett

Corresponding Author René Karadakic.
E-mail: rene.karadakic@mtec.ethz.ch

This PDF file includes:

Supporting text

Supporting Information Text

Construction of the Chemotherapy Cohort from Medicare Claims

We used 100% national Medicare fee-for-service (FFS) claims to construct a cohort of beneficiaries initiating infused or injected chemotherapy between 2017 and 2021. Data were extracted from the Carrier, Outpatient, and Inpatient claims files, as well as the Master Beneficiary Summary File (MBSF) and the Chronic Conditions Data Warehouse (CCW). All analyses were conducted in the Centers for Medicare & Medicaid Services (CMS) Virtual Research Data Center (VRDC).

Identification of Chemotherapy Claims. We combined line-level data from the Carrier and Outpatient revenue files (2016–2021) to identify all Part B chemotherapy claims. Claims were restricted to those containing a chemotherapy Healthcare Common Procedure Coding System (HCPCS) code (including immune checkpoint inhibitors (ICI) and all other cancer-related infused or injected drugs). Each claim was linked to diagnosis codes to verify the presence of a cancer diagnosis, using International Classification of Diseases ICD-10 mappings from an external crosswalk. For claims with multiple diagnosis codes, the first valid cancer diagnosis was retained.

Chemotherapy Initiation Episodes. We defined a new chemotherapy initiation as the first Part B chemotherapy claim after at least 360 days without any chemotherapy administration. For each beneficiary, only the first qualifying claim within a 12-month window was retained. We created an indicator for ICI use based on HCPCS codes for ICI drugs available during the study period (atezolizumab, avelumab, cemiplimab, durvalumab, ipilimumab, nivolumab, pembrolizumab).

Vaccination Exposure. Vaccination exposure was defined from Part B claims using HCPCS codes for SARS-CoV-2 mRNA vaccines (BioNTech/Pfizer and Moderna), adenoviral vaccine (Janssen/Johnson & Johnson), and influenza vaccines. For each beneficiary, the vaccination date closest to chemotherapy initiation was identified, and the interval in days between vaccination and treatment initiation was calculated. Separate indicators were created for mRNA COVID-19, adenoviral COVID-19, and influenza vaccination occurring within 100 days of systemic anticancer therapy initiation. Consequently, beneficiaries receiving more than one vaccine type within the exposure window contributed to each corresponding vaccine-specific analysis. To enable direct comparison of the two COVID-19 vaccine platforms, recipients of Janssen vaccination were excluded from analyses of mRNA vaccination, and recipients of mRNA vaccination were excluded from analyses of Janssen vaccination. Influenza vaccination was analyzed independently and was not used as an exclusion criterion for the COVID-19 vaccine analyses. Results were robust when restricting the exposure definition to vaccination occurring within 100 days prior to systemic anticancer therapy initiation, reducing potential immortal time bias.

Beneficiary Characteristics. Demographic characteristics (age, sex, race and ethnicity, disability status, dual eligibility, and low-income subsidy months) were derived from the Master Beneficiary Summary File (MBSF). Continuous Part A and B enrollment without Medicare Advantage participation was identified using monthly indicators. Chronic condition indicators (28 CCW conditions) were merged by year from the MBSF-CC files, and a count of chronic conditions was created for comorbidity adjustment. The primary outcome was all-cause mortality following treatment initiation, obtained from the Medicare Master Beneficiary Summary File.

Final Cohort. All files were linked by beneficiary ID and year. The final dataset includes claim-level identifiers, vaccination status, demographic and clinical characteristics, and cancer type assignments. The final sample for our analysis was then restricted to only include patients initiating chemotherapy between 09/01/2020 and 06/30/2021.

The analytic sample included 121,676 Medicare beneficiaries initiating systemic anticancer therapy, including 10,824 initiating immune checkpoint inhibitor (ICI) therapy and 110,852 initiating non-ICI therapy. Mean age was similar across cohorts (74.4 years for ICI recipients and 74.9 years for non-ICI recipients). Women represented 41.1% of the ICI cohort and 31.6% of the non-ICI cohort. Black beneficiaries represented 8.6% of ICI recipients and 4.1% of non-ICI recipients, while Hispanic beneficiaries represented 1.3% in both groups. Mean numbers of chronic conditions were 6.7 among ICI recipients and 6.0 among non-ICI recipients. Prior influenza vaccination was common in both cohorts (49.9% and 49.8%, respectively). Among ICI recipients, the most common cancer types were lung cancer (35.3%), skin cancer (19.2%), and prostate/genitourinary cancers (17.7%). Within 100 days of treatment initiation, 2,844 ICI recipients received mRNA COVID-19 vaccination, 117 received adenoviral COVID-19 vaccination, 2,602 received influenza vaccination, and 5,261 had no recorded vaccination exposure within the study window.

Model Specification

Cox Proportional Hazard Models. We estimated the association between vaccination and survival using the following Cox proportional hazards specification:

$$h_i(t) = h_0(t) \exp\left(\beta_1 \text{Vaccine}_i + \beta_2 X_i + \gamma_{c(i)} + \delta_{ym(i)}\right)$$

where $h_i(t)$ denotes the hazard of death at time t for individual i, and $h_0(t)$ denotes the baseline hazard. Vaccine_i is an indicator for receipt of a vaccine within 100 days of chemotherapy initiation (mRNA COVID-19, adenoviral COVID-19, or influenza vaccination). X_i includes demographic and clinical covariates, including indicators for race and ethnicity, disability

status, continuous fee-for-service enrollment, categorical controls for months receiving low-income subsidy and dual eligibility, an indicator for prior influenza vaccination, and chronic condition indicators from the Chronic Conditions Data Warehouse. $\gamma_{c(i)}$ denotes cancer-type fixed effects and $\delta_{ym(i)}$ denotes year-by-month fixed effects for treatment initiation. Standard errors were clustered at the cancer-type level.

Linear Probability Models. We estimated the interaction between vaccination and immune checkpoint inhibitor (ICI) initiation using the following linear probability model:

$$Y_i = \alpha + \beta_1 \text{ICI}_i + \beta_2 \text{Vaccine}_i + \beta_3 \left(\text{ICI}_i \times \text{Vaccine}_i\right) + \beta_4 X_i + \gamma_{c(i)} + \delta_{ym(i)} + \varepsilon_i$$

where Y_i is an indicator for mortality within a given follow-up interval; ICI_i is an indicator for initiation of immune checkpoint inhibitor therapy; and Vaccine_i is an indicator for receipt of a vaccine within 100 days of treatment initiation (mRNA COVID-19, adenoviral COVID-19, or influenza vaccination). The interaction term $\text{ICI}_i \times \text{Vaccine}_i$ captures whether the association between vaccination and mortality differs between ICI and non-ICI recipients.

X_i includes demographic and clinical covariates, including indicators for race and ethnicity, disability status, continuous fee-for-service enrollment, categorical controls for months receiving low-income subsidy and dual eligibility, an indicator for prior influenza vaccination, and chronic condition indicators from the Chronic Conditions Data Warehouse. $\gamma_{c(i)}$ denotes cancer-type fixed effects and $\delta_{ym(i)}$ denotes year-by-month fixed effects for treatment initiation. Standard errors were clustered at the cancer-type level.
